# Comparison of two skin temperature assessment methods after the application of topical revulsive products: Conductive iButton data logger system vs contact‐free infrared thermometry

**DOI:** 10.1111/srt.12847

**Published:** 2020-04-09

**Authors:** Rahel Stoop, Erich Hohenauer, Dirk Aerenhouts, André O. Barel, Tom Deliens, Ron Clijsen, Peter Clarys

**Affiliations:** ^1^ Department of Business Economics, Health and Social Care University of Applied Sciences and Arts of Southern Switzerland Landquart Switzerland; ^2^ Department of Movement and Sport Sciences Faculty of Physical Education and Physiotherapy Vrije Universiteit Brussel Brussels Belgium; ^3^ International University of Applied Sciences THIM Landquart Switzerland; ^4^ School of Sport, Health and Exercise Science University of Portsmouth Portsmouth UK; ^5^ Department of Health Bern University of Applied Sciences Berne Switzerland

**Keywords:** infrared thermography, laser speckle contrast imaging, revulsive products, skin temperature, thermocouples

## Abstract

**Background:**

Skin temperature assessments comprise conductive and contact‐free techniques. Comparison between conductive data loggers and contact‐free thermometry after the application of revulsive products is scarce. This study aimed to compare iButton data loggers with an infrared thermometer after the application of two revulsive products. Secondly, the relation between skin temperature kinetics with skin's perfusion of microcirculation was investigated.

**Materials and methods:**

Healthy females (n = 25) were randomly allocated to two groups, representing the products A and B. Skin temperature was measured with “iButtons” and an infrared pistol at baseline and up to 1 hour after application. Skin's perfusion of microcirculation was monitored with a laser speckle contrast imager.

**Results:**

Baseline “iButton” temperature values were significantly lower compared with infrared pistol values in both groups. After application of the products, skin temperature decreased as recorded with both devices followed by an increase to baseline values when measured with the pistol. The results obtained by the “iButtons” reached values above baseline in both products towards the end of the follow‐up period. A moderate correlation was found between infrared pistol and “iButton” system in product A, with a weak negative correlation between skin's perfusion of microcirculation and temperature devices. For product B, the correlation between the devices was moderate and between skin's perfusion and temperature devices weak and positive.

**Conclusion:**

Both devices produced similar kinetics, except at baseline, where they may differ as metallic loggers have been insufficiently adapted to skin temperature. Skin's perfusion of microcirculation could not explain skin temperature changes.

## 1 INTRODUCTION

Skin temperature measurement techniques comprise conductive thermocouples, thermistors and telemetry systems as well as contact‐free infrared thermometry and imaging.^1^, ^2^, ^3^, ^4^ The measurement is challenging even more when sweat or topical products cover the skin surface.^5^, ^6^, ^7^ The ingredients of plant‐derived revulsive products may induce changes in skin blood flow, affecting skin temperature.^8^, ^9^, ^10^, ^11^ To the best of authors’ knowledge, to date, no study has compared conductive and contact‐free skin temperature measurement methods to perform a continuous observation of the physiological changes induced by revulsive products.

Therefore, the aim of this study was (a) to compare skin temperature results of the conductive iButton data logger system with the contact‐free infrared pistol at each time point from baseline to 60‐minutes follow‐up, (b) to investigate skin temperature changes within each device and product, and (c) to measure skin's perfusion of microcirculation to evaluate its relation with skin temperature changes.

## 2 MATERIALS AND METHODS

### 2.1 Study design and participants

This study was approved by the Swiss Cantonal Ethical Committee of Zurich, KEK‐ZH ID 2016‐01541, in accordance with the Declaration of Helsinki (ICH‐GCP). Twenty‐six young healthy Caucasian female volunteers were recruited. After written informed consent, the participants were checked for inclusion and exclusion criteria. The included females were non‐smokers, aged between 18 and 35 years with healthy skin conditions. They were randomly allocated to one of the experimental groups (A treated with product A or B treated with product B) by drawing lots. The products were applied on pre‐defined areas on the lumbar back region. Demographics of the participants and environmental conditions are presented in Tables 1 and 2.

**Table 1:** Participants’ characteristics grouped by intervention product (mean ± SD)

	Age (y)	Height (m)	Weight (kg)	BMI (kg/m^2^)
Group A (n = 13)	24.4 ± 4.5	1.67 ± 0.04	64.7 ± 8.1	23.2 ± 2.7
Group B (n = 12)	23.9 ± 3.9	1.68 ± 0.08	68.7 ± 11.3	24.4 ± 3.2

Note

A, B = intervention product, n = number of participants, none of the variables were significantly different between group A and B at alpha = 0.05.

**Table 2:** Environmental conditions grouped by intervention product (mean ± SD)

	Room temperature at start (°C)	Room temperature at end (°C)	Relative humidity at start (%)	Relative humidity at end (%)
Group A (n = 13)	22.8 ± 1.5	23.3 ± 1.3	39.5 ± 0.8	39.5 ± 0.9
Group B (n = 12)	23.3 ± 1.0	23.5 ± 1.0	39.5 ± 1.1	39.2 ± 1.3

Note

°C = degrees of Celsius, none of the variables were significantly different between group A and B at alpha = 0.05.

### 2.2 Interventional products

Axanova hot gel^®^ was chosen as product A and Dolor‐X hot gel^®^ as product B. Both products are over‐the‐counter products in Switzerland. Detailed information on the concentration of the components was not available.

### 2.3 Measurements

Skin temperature was conductively assessed with a telemetric metallic thermochronic data logger system (iButton DS1922L‐F5, Maxim Integrated Products). The “iButtons” were coded with the appertaining interface on a laptop computer to measure skin temperature in 1‐second intervals with the highest achievable resolution of 0.0625°C for 11 bit.^12^ After finishing the measurements, they were connected with the appertaining interface on a laptop computer for data collection. Further, skin temperature was measured contact‐free by a handheld infrared pistol providing a resolution of 0.1°C (Voltcraft IR 800‐20D IR Thermometer).^13^ The pistol emits two separate laser light beams and captures the reflecting light by a diode. The appropriate measurement distance rectangularly to the skin surface was reached once both aiming beams united to one light spot. The digitally displayed temperature value was manually transferred on the data sheet.

Skin's perfusion of microcirculation measurement was performed as described elsewhere.^14^

### 2.4 Experimental setting

**Figure 1:**
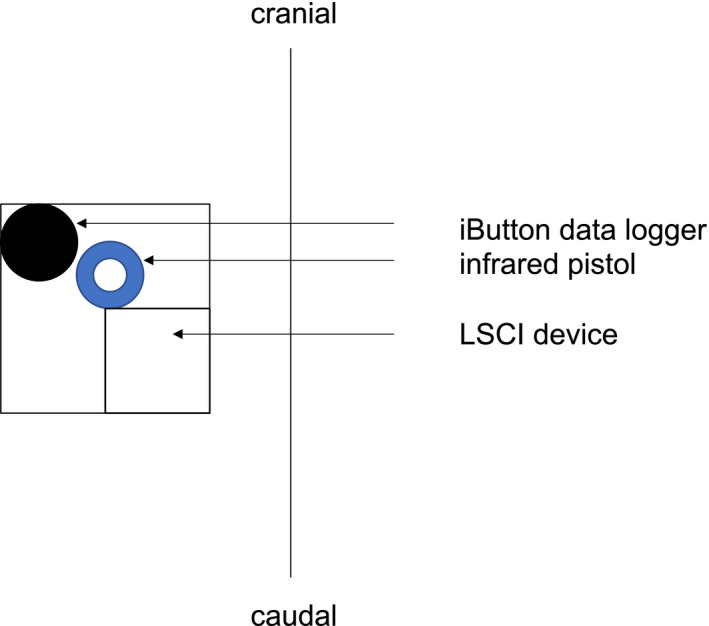
Experimental setting on the lumbar back region

Room temperature and humidity (RH) were monitored by a multimeter (Voltcraft MT52) and kept constant between 22.5 to 23.5°C and 39%‐40% RH throughout all measurements. The participants were advised to refrain from drinking any caffeine‐containing beverages at least 24 hours before the start of the experiment, not to shower, not to apply any body lotion and to avoid any exhaustive exercise prior to the measurements. After arriving in the laboratory, they changed into shorts and unclothed their upper body up to the underwear. Afterwards, the participants laid down in prone position on a therapeutic plinth. One side of the lumbar back was randomly defined as application area. A 10 × 10 cm investigational area was defined as region of interest (ROI) and confined with elastic tape strips. The “iButton” was placed on its foreseen place as shown in Figure 1. Afterwards, the acclimatization period of 20 minutes started where the participants were advised to avoid any movements.

Afterwards, baseline measurements started. Firstly, skin's perfusion of microcirculation was assessed followed by skin temperature with the infrared pistol. After completion of the baseline measurements, the “iButton” was removed and the product A or B was applied on the participant pre‐defined lower back region. Exactly, 0.5 g (Kern 770 precision scale)^15^ of the investigational product A or B was applied with circular movements using the one‐finger‐glove technique.^16^ The participants and the investigators were blinded to the products. After reaching the maximum absorption capacity of the skin, the “iButton” was re‐applied and the post‐application measurement (T0) started. Follow‐up measurements were conducted in 5‐minute intervals up to 40 minutes (T5‐T40), then in 10‐minute intervals up to 1 hour (T50, T60).

### 2.5 Statistical analysis

Data were analysed using the Statistical Package for Social Sciences (SPSS), version 25.0 (IBM Corporation). Descriptive statistics (means ± standard deviations [SD]) were retrieved. Independent samples *t* tests were applied to test for differences in characteristics and environmental conditions between group A and B. Data were analysed and presented as absolute values for skin temperature and normalized changes from baseline values were used for skin's perfusion of microcirculation. The ROIs (A, B) were each analysed separately by a Two‐way Repeated Measures ANOVA with both “time” and “device” as within factors to assess changes over time (baseline, T0, T5, T10, T15, T20, T25, T30, T35, T40, T50, T60) and differences in skin temperature measurement technique (“iButtons,” infrared pistol), respectively, as well as the interaction effect between both. One‐way Repeated Measures ANOVAs were used to analyse changes over time in skin temperature measurement techniques “iButtons” and infrared pistol, respectively (baseline, T0, T5, T10, T15, T20, T25, T30, T35, T40, T50, T60). Pearson's correlations were performed to assess the relationship between the two skin temperature measurement techniques on the one hand and between skin temperature measurement techniques and skin perfusion on the other hand for product A and B, respectively. A One‐way Repeated Measures ANOVA was performed to detect changes over time in skin's perfusion (baseline, T0, T5, T10, T15, T20, T25, T30, T35, T40, T50, T60). *P*‐values < .05 were considered as statistically significant. Bonferroni corrected post hoc paired samples *t* tests were used to detect differences between baseline and the different time points (so, *P*‐values < .0045 were considered as statistically significant), as well as differences between both techniques per time point (so, *P*‐values < .0042 were considered as statistically significant).

## 3 RESULTS

Participants’ age, height, weight and BMI, as well as environmental conditions, were comparable in both groups (all *P* > .05; see Tables 1 and 2).

**Figure 2:**
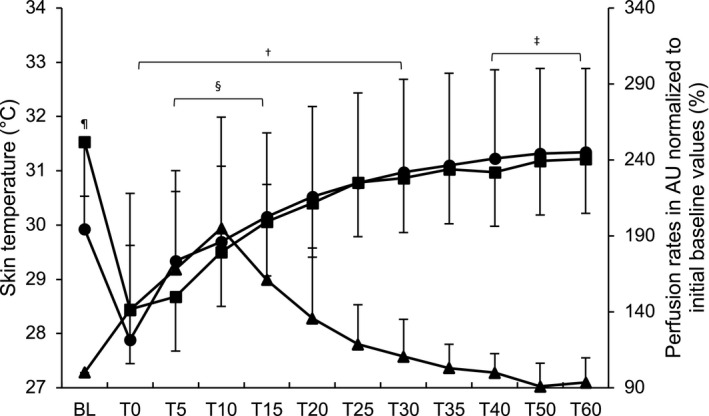
Skin temperature, measured by the conductive iButton data logger system and the contact‐free infrared pistol, with skin's perfusion of microcirculation of product A over time. Legend: ▬● “iButton,” ▬■ infrared pistol, ▬▲ skin's perfusion of microcirculation, AU = arbitrary units, ^†^ *P* < .05 infrared pistol within difference compared to baseline, ^‡^ *P* < .05 “iButton” within difference compared to baseline, ^§^ *P* < .05 skin's perfusion of microcirculation within difference compared to baseline, ^¶^ *P* < .05 between difference “iButton” vs infrared pistol

In group A, no interaction effect was found for skin temperature (*F*_[11,2]_ = 7.204, *P* = .128, $\eta_{\text{partial}}^{2}$ = 0.975). A significant main effect for time (*F*_[11,2]_ = 54.016, *P* = .018, $\eta_{\text{partial}}^{2}$ = 0.997), and no effect for device (*F*_[1,12]_ = 0.031, *P* = .863, $\eta_{\text{partial}}^{2}$ = 0.003) was reported. Both skin temperature devices differed significantly at baseline (*P* < .001). The “iButtons” and the infrared pistol measured equal values at the remaining time points. At T0, significantly lower skin temperatures were reported when measured by the “iButtons” compared to baseline (*P* = .001) and higher values were found for T40, T50 and T60 (*P* = .004, *P* = .002, *P* = .002, respectively). Skin temperature measurements by the infrared pistol showed significantly lower skin temperature results compared with baseline at T0, T5, T10, T15, T20, T25 (all *P* ≤ .001) and T30 (*P* = .001). A correlation of *r* = .820 (*P* < .001) was found between both temperature measurement devices. Although skin's perfusion of microcirculation showed no main effect for time (*F*_[11,2]_ = 1.443, *P* = .479, $\eta_{\text{partial}}^{2}$ = 0.888), further analyses per time point revealed significantly higher results compared with baseline at T5, T10 and T15 (*P* = .003, *P* = .001, *P* = .004, respectively; see Figure 2). A correlation of *r* = −.175 (*P* = .028) and *r* = −.208 (*P* = .009) was found between skin perfusion and skin temperature as measured with the “iButtons” and the infrared pistol, respectively.

**Figure 3:**
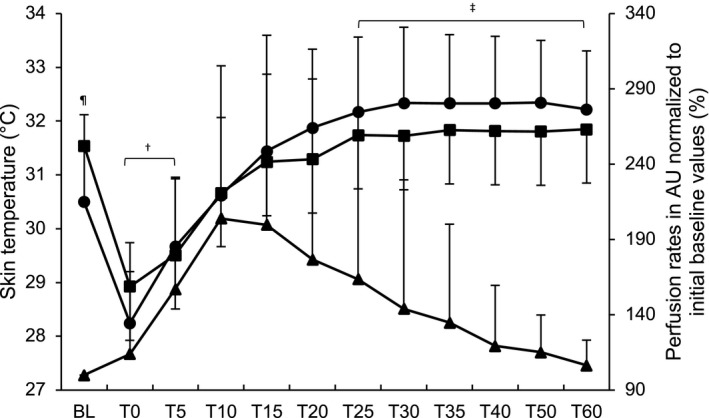
Skin temperature, measured by the conductive iButton data logger system and the contact‐free infrared pistol, with skin's perfusion of microcirculation of product B over time. Legend: ▬● “iButton,” ▬■ infrared pistol, ▬▲ skin's perfusion of microcirculation, AU = arbitrary units, ^†^ *P* < .05 infrared pistol within difference compared to baseline, ^‡^ *P* < .05 “iButton” within difference compared to baseline, ^¶^ *P* < .05 between difference “iButton” vs infrared pistol

In group B, no interaction effect was detected for skin temperature (*F*_[11,1]_ = 0.569, *P* = .788, $\eta_{\text{partial}}^{2}$ = 0.862). No overall main effects for time (*F*_[11,1]_ = 18.233, *P* = .181, $\eta_{\text{partial}}^{2}$ = 0.995), nor device (*F*_[1,11]_ = 0.570, *P* = .466, $\eta_{\text{partial}}^{2}$ = 0.049) were found. The two skin temperature devices displayed significantly different values at baseline (*P* = .001). The results of all other time points indicated that both devices obtained comparable skin temperature values. The “iButtons” measured significantly lower skin temperatures compared with baseline at T0, T25, T30, T35, T40, T50, T60 (*P* < .001, *P* = .004, *P* = .002, *P* = .002, *P* = .001, *P* = .002, *P* = .002, respectively). Skin temperature values obtained by the infrared pistol were significantly lower compared with initial values at T0 and T5 (both *P* < .001). A correlation of *r* = .777 (*P* < .001) was found between both temperature measurement devices. No effect for time was reported for skin's perfusion of microcirculation (*F*_[11,1]_ = 2.398, *P* = .468, $\eta_{\text{partial}}^{2}$ = 0.963), and no significant changes compared with baseline values were observed at any time point (all *P* > .05; see Figure 3). A correlation of *r* = .276 (*P* = .001) and *r* = .202 (*P* = .015) was found between skin perfusion and skin temperature as measured with the “iButtons” and the infrared pistol, respectively.

## 4 DISCUSSION

The aim of this study was (a) to compare skin temperature results of the conductive “iButtons” with the contact‐free infrared pistol at each time point of interest from baseline to 60‐minutes follow‐up, (b) to investigate skin temperature changes within each device and product, and (c) to measure skin's perfusion of microcirculation to evaluate its relation with skin temperature changes. The main results showed that for both products the conductive and the contact‐free skin temperature devices differed significantly at baseline and provided equal values at every other observed time point. Kinetics of skin temperature and of skin's perfusion of microcirculation differed and showed weak and non‐significant correlations.

Both methods are regarded as suitable to measure skin temperature after the application of a revulsive product since the average values per time point were similar. Nevertheless, a correlation between both techniques in each product indicates a certain degree of random deviation at the individual level. Skin temperature difference between the observed two measurement methods occurred at baseline with untreated skin conditions. A plausible explanation for this result could be that the adaptation time of 20 minutes used was insufficient to equalize the metallic shell cover of the “iButtons” with the skin surface.^17^ During the application time of the products, the “iButtons” were placed on a table at room temperature, which might have allowed the “iButtons” to lose temperature and re‐adjust to room conditions. Following Pinnagoda et al,^18^ thermistors should be stored on an untreated blank skin area.

Apart from the initial drop in skin temperature (evaporation of alcohol), the two devices detected changes of skin temperature to the initial values at different time points. Whilst the infrared technique detected lower skin temperature compared to baseline in the first half of the follow‐up time, the conductive device measured higher values compared to baseline towards the end. This finding, apparent in both products, could be explained by the creation of a microclimate between skin surface and “iButtons.”^19^

Evaporation of the volatile components of the applied products induced a delay between the increase of skin's perfusion of microcirculation and skin temperature. Further, skin temperature remained elevated whilst values of perfusion of microcirculation already returned to baseline. As consequence, weak correlations between skin's perfusion of microcirculation and skin temperature were found in both products. The LSCI‐device used in our study detects skin's perfusion of microcirculation of the superficial skin layers up to the capillary loops, not assessing underlying regions, whereas skin temperature might be affected by vasodilation of capacity vessels in deep skin areas.^20^, ^21^

After the initial skin temperature drop, skin temperature kinetics paralleled with skin's perfusion of microcirculation until its peak. Skin's perfusion of microcirculation reached significant changes compared with baseline only in product A, that is at 5 up to 15 minutes post‐application. The onset of increase in perfusion level started directly after the products’ application. This might indicate a fast penetration through the stratum corneum, allowing the product to reach the vascular bed, locally inducing vasodilation.^8^ Controversially, Kotaka et al^11^ showed a concentration‐dependent time latency of 1 to 4 minutes of skin blood flow increase after the application of camphor. In contrary to the current study, they applied the product without rubbing. Therefore, the perfusion kinetics of our study could be related to the rubbing effect induced by the application. Further, they used a total quantity of product that was 93% higher and in a different composition than in the present study. These two factors might explain their long‐lasting increase in skin blood flow up to 50 minutes post‐application,^11^ whereas the findings of our study showed a steady decrease after a (non‐significant) peak around 10 minutes. Besides, other studies using menthol gel showed an increase in skin's perfusion of microcirculation.^8^, ^22^ Therefore, the composition of the products used and the small quantity of ointment applied in comparison with former studies might explain the differences between the results on skin's perfusion of microcirculation.

The authors like to address some suggestions for upcoming studies. Randomizing the ROIs would allow to control for the possible regional micro‐vessel density differences,^23^ maybe influencing skin temperature. Secondly, the authors suggest adjusting the “iButtons” longer than 20 minutes and to store them on a neighbouring skin region.^18^

## 5 CONCLUSION

Conductive iButton data logger system and contact‐free infrared thermometry give similar kinetics of skin temperature after the application of revulsive products. Contact‐free infrared thermometry might be more suitable compared with the conductive iButton data logger system in terms of initial adaptation time to skin temperature and covering induced disturbances.
